# Peer collaborative learning and academic engagement in university libraries: a moderated mediation model and latent profile analysis

**DOI:** 10.3389/fpsyg.2025.1759026

**Published:** 2026-01-16

**Authors:** Hanqiao Tang, Lei Shen

**Affiliations:** 1 School of Education, Huainan Normal University, Huainan, China; 2 School of Finance and Mathematics, Huainan Normal University, Huainan, China

**Keywords:** academic engagement, basic psychological needs, latent profile analysis, peer collaborative learning, self-determination theory

## Abstract

**Objective:**

As university libraries transform into “Learning Commons,” peer collaborative learning has become increasingly common. However, the complexity of its effectiveness and its underlying mechanisms remain underexplored. This study systematically investigates the relationship between peer collaborative learning and academic engagement. Integrating both person-centered and variable-centered approaches, the study employs Latent Profile Analysis (LPA) to identify heterogeneous groups of students based on their collaboration patterns and engagement levels, challenging the conventional wisdom that “collaboration is always beneficial.” Concurrently, based on Self-Determination Theory (SDT), a mediation model is constructed to test the bridging role of basic psychological needs (competence and relatedness).

**Methods:**

Using a questionnaire survey, data were collected from 820 university students to measure their quality of peer collaborative learning, basic psychological need satisfaction, and academic engagement. The SPSS PROCESS macro was used for mediation analysis, and Mplus was used for Latent Profile Analysis.

**Results:**

(1) LPA identified four heterogeneous profiles: “High-Achieving All-Rounders” (26.1%), “Balanced Developers” (25.5%), “Inefficient Socializers” (27.6%), and “Indifferent and Unengaged” (20.9%). Notably, the largest group, “Inefficient Socializers,” exhibited a distinct pattern of “high emotional support, low academic engagement.” (2) Basic psychological needs played a significant partial mediating role in the relationship between the quality of peer collaborative learning and academic engagement, with the mediating effect accounting for 52.3% of the total effect. (3) The moderating effect of academic discipline was not significant.

**Conclusion:**

The study confirms that while high-quality peer collaboration can promote academic engagement by satisfying students' psychological needs, not all forms of collaboration are beneficial. The discovery of the “Inefficient Socializers”—the largest profile—is the core contribution of this research. It exposes the existence of a “pseudo-collaboration” trap, where social interaction detached from task-oriented goals may actually inhibit academic engagement. This finding offers crucial practical implications for the design of university learning spaces (shifting from “promoting co-presence” to “fostering effective interaction”) and for academic advising (enabling the precise identification and intervention for different student types).

## 1 Introduction

### 1.1 Research background and problem statement

Amidst the global wave of higher education reform, university libraries are undergoing a profound transformation from traditional “book repositories” to modern “Learning Commons” (Chaddha and Kanjilal, 2022). This shift represents not merely a physical restructuring but an evolution in educational philosophy (Fagan et al., 2021), with its core advocating for student-centered, active, and collaborative learning models. In this context, Peer Collaborative Learning has become the most prevalent activity within Learning Commons, especially as hybrid learning models have gained prominence in the post-pandemic era, making both online and offline collaboration equally vital (Fabriz et al., 2021). Students are no longer isolated recipients of knowledge but are co-constructing knowledge and solving problems through interaction, discussion, and mutual support with their peers.

Theoretically, high-quality peer collaboration is widely believed to yield numerous academic benefits, such as enhancing critical thinking, improving problem-solving skills, and fostering academic engagement (Li, 2025). However, the reality is far more complex than theory suggests. A walk through the collaborative zones of any university library reveals starkly different scenes: some groups are engaged in heated, focused discussions; others exude a relaxed atmosphere, but their conversations seem unrelated to academics; still others, though physically in a group, appear disconnected and disengaged. This observation raises a fundamental practical question: Does simply organizing students to learn “together” necessarily promote their academic engagement? If not, what are the key factors that determine the effectiveness of collaborative learning? And what are the underlying psychological mechanisms?

Much of the existing research has focused on confirming the positive relationship between “collaboration” and “outcomes” (Li and Xue, 2023), while largely neglecting the “black box” of the collaborative process itself. Specifically, how do peer interactions translate into individual learning motivation? Why does the same collaborative setting inspire academic passion in some students but have the opposite effect on others? Self-Determination Theory (SDT) provides a powerful theoretical lens to open this “black box.” The theory posits that an individual's intrinsic motivation and positive behaviors stem from the satisfaction of three basic psychological needs: Competence, Relatedness, and Autonomy (Reeve and Cheon, 2021). Perhaps it is by fulfilling these deep-seated psychological needs that peer collaboration ultimately influences student academic engagement.

Furthermore, students are not a homogeneous group. They enter collaborative situations with different personality traits, learning habits, and social needs. Traditional variable-centered approaches, which yield an “average effect,” may obscure the unique response patterns of different student subgroups (Olivier et al., 2025). For instance, are there students for whom collaboration actually inhibits academic engagement? Identifying these heterogeneous groups is crucial for providing targeted and personalized academic guidance.

### 1.2 Research objectives and significance

Based on the foregoing, this study aims to systematically explore the complex relationship between peer collaborative learning and academic engagement within the context of university library Learning Commons. The specific research objectives are:

To test the mediating mechanism (H1): Based on Self-Determination Theory, to examine whether the two basic psychological needs of competence and relatedness act as mediators in the relationship between the quality of peer collaborative learning and academic engagement.

To explore boundary conditions (H2): To investigate whether academic discipline (Humanities vs. STEM) acts as a moderator in the aforementioned mediation pathway, i.e., whether there are differences in the psychological need satisfaction that students from different disciplines derive from peer collaboration.

To identify heterogeneous groups (H3): To adopt a person-centered research method (Latent Profile Analysis) to identify distinct latent profiles of students based on their “collaboration patterns-engagement levels” and to analyze the characteristics of each profile.

The theoretical significance of this study is two-fold. First, by introducing SDT into the context of collaborative learning in libraries, it uncovers the “social interaction → psychological satisfaction → academic behavior” pathway, deepening the understanding of the intrinsic mechanisms of collaborative learning. Second, by employing Latent Profile Analysis, it addresses the concern for student heterogeneity and challenges the monolithic view that “collaboration is always beneficial,” providing empirical evidence for the refinement of related theories.

The practical significance is more direct. The findings will offer a scientific basis for the design and management of learning spaces in university libraries, for instance, by informing how to design spaces that better promote “effective interaction.” Simultaneously, by identifying specific student groups like the “Inefficient Socializers,” this study can provide targeted intervention points for academic advising centers and counselors, helping students optimize their collaborative strategies and thereby truly maximizing the value of the Learning Commons.

## 2 Literature review and research hypotheses

### 2.1 Peer collaborative learning and its quality

Peer collaborative learning is defined as a process where “students work together in small groups to complete a learning task, during which they share information, support each other, and share responsibility” (Song et al., 2024). Its core lies in interaction. However, the quantity of interaction does not necessarily equate to its quality. Storch (2002) proposed a two-dimensional framework for assessing the quality of collaborative interaction: first, the task dimension, which refers to the extent to which group members focus on the learning task itself, including asking questions, explaining concepts, and constructing arguments—what we term task-oriented interaction. Second, the relational dimension, which refers to the mutual respect, encouragement, and emotional support displayed among members—what we term socio-emotional support. High-quality collaborative learning should be an organic combination of these two dimensions. A large body of research has shown that high-quality peer collaboration significantly predicts higher academic achievement and stronger learning motivation.

### 2.2 Academic engagement

Academic engagement is a key indicator of the quality of student learning and is considered a more stable and developmentally significant predictor than academic performance (Estévez et al., 2021). Schaufeli et al. (2006) define it as a positive, fulfilling, learning-related state of mind characterized by three dimensions:

Vigor: High levels of energy, mental resilience, and persistence in learning.

Dedication: A strong sense of enthusiasm, pride, and significance derived from learning.

Absorption: Being fully engrossed in learning, to the point of losing track of time.

Numerous studies have confirmed that peer learning interactions are a key antecedent of academic engagement (Letchumanan et al., 2025). This study adopts this three-dimensional definition, treating academic engagement as the core outcome variable for measuring the effectiveness of collaborative learning.

### 2.3 The mediating role of self-determination theory and basic psychological needs

Self-Determination Theory (SDT) is a core theory explaining human motivation (Chiu, 2022). It posits that all human behaviors are driven by three basic psychological needs:

Need for Competence: The need to feel effective in one's interactions with the environment and to experience opportunities to express one's capacities.

Need for Relatedness: The need to establish secure and warm connections with others and to feel accepted and cared for by a group.

Need for Autonomy: The need to feel that one's actions are volitional and congruent with one's sense of self.

While the need for autonomy is equally crucial, this study deliberately focuses on competence and relatedness. This decision is based on the specific context of informal, often spontaneously formed collaborative learning in settings like libraries. In such environments, the most direct and salient psychological experiences are often related to the cognitive challenges of the task (competence) and social acceptance by peers (relatedness). The element of autonomy—feeling one's actions are volitional—is certainly at play, but it can be more complex to isolate in group settings where consensus and shared direction are necessary. By focusing on competence and relatedness, we aim to deeply analyze the core socio-psychological processes of collaborative interaction. We acknowledge that this is a simplification and the omission of autonomy might lead to an incomplete picture of the motivational dynamics. For example, a lack of perceived autonomy within a group could thwart motivation even if competence and relatedness needs are met. Thus, incorporating autonomy into a more comprehensive model remains an important and necessary direction for future research (Guo et al., 2025).

SDT maintains that when the environment supports and satisfies these basic psychological needs, an individual's intrinsic motivation and positive behaviors (such as academic engagement) are fostered (Reeve and Cheon, 2021). In recent years, this theory has been widely applied to explain academic motivation and engagement in various learning contexts (Chiu, 2021; Shen et al., 2025; Yang et al., 2025). We apply this logic to the peer collaboration context:

When students engage in task-oriented interactions (e.g., solving problems together, discussing difficult concepts), they can leverage their peers' knowledge to overcome challenges they couldn't tackle alone. This “I can do it” experience directly satisfies their need for competence (Shin and Bolkan, 2021). When students receive socio-emotional support from peers (e.g., encouragement, comfort, validation), they feel accepted and cared for by the team, thereby satisfying their need for relatedness (Karabacak-Çelik and Aşantugrul, 2024; Zhang and Qian, 2024). According to SDT, these two satisfied psychological needs will ultimately translate into greater learning “vigor,” a stronger sense of “dedication,” and deeper “absorption” (Li, 2025). Based on this, we propose our core mediation hypothesis:

H1: Basic psychological needs mediate the relationship between the quality of peer collaborative learning and academic engagement.

H1a: The need for competence mediates the relationship between task-oriented interaction and academic engagement.

H1b: The need for relatedness mediates the relationship between socio-emotional support and academic engagement.

### 2.4 The moderating role of academic discipline

Different academic disciplines have distinct learning tasks and thinking paradigms. The humanities (e.g., literature, history) often involve open-ended discussions and critical thinking, whereas STEM disciplines focus more on logical deduction and solving standardized problems. This difference might affect the patterns and effectiveness of peer collaboration. It must be acknowledged that a simple “humanities vs. STEM” dichotomy is a somewhat crude classification that may not capture the full complexity within and across disciplines. Nevertheless, it serves as a common and meaningful starting point for exploring macro-level differences. For instance, STEM students might experience greater competence satisfaction from task-oriented interactions due to the quick attainment of definitive answers. In contrast, humanities students might be more sensitive to their need for relatedness during socio-emotional interactions, as they may require more emotional resonance and acceptance of their viewpoints. Thus, academic discipline could act as a boundary condition, moderating the strength of the aforementioned mediation paths. Accordingly, we propose the following moderation hypothesis:

H2: Academic discipline moderates the relationship between peer collaboration and basic psychological needs.

H2a: Compared to humanities students, the positive association between task-oriented interaction and the need for competence is stronger for STEM students.

H2b: Compared to STEM students, the positive association between socio-emotional support and the need for relatedness is stronger for humanities students.

### 2.5 Heterogeneity in student participation and latent profile analysis

Traditional “variable-centered” methods assume a homogeneous sample and aim to find “average” relationships between variables. However, this approach can mask unique patterns within different subgroups of individuals. For example, is there a group of students who turn collaborative learning into a purely social activity, leading to a decline in academic engagement? Is there another group that maintains high engagement even without high-quality collaboration?

To answer these questions, this study introduces the “person-centered” approach of Latent Profile Analysis (LPA). LPA is a statistical technique that identifies several mutually exclusive, internally homogeneous latent classes within a sample based on individuals' response patterns across a series of continuous variables. With LPA, we can move beyond the question of “What is the relationship between variables?” to a deeper exploration of “What types of students exist?” In recent years, this method has been widely used to identify heterogeneous student groups in educational psychology (Gaete Sepúlveda et al., 2025; Huttunen et al., 2025; Liao et al., 2023; Olivier et al., 2025; Zhao, 2024). This helps us uncover complex, non-linear, and non-mainstream relationship patterns. Therefore, we propose an exploratory hypothesis:

H3: Several heterogeneous latent profiles of students exist based on their peer collaboration patterns and academic engagement levels.

**Figure 1:**
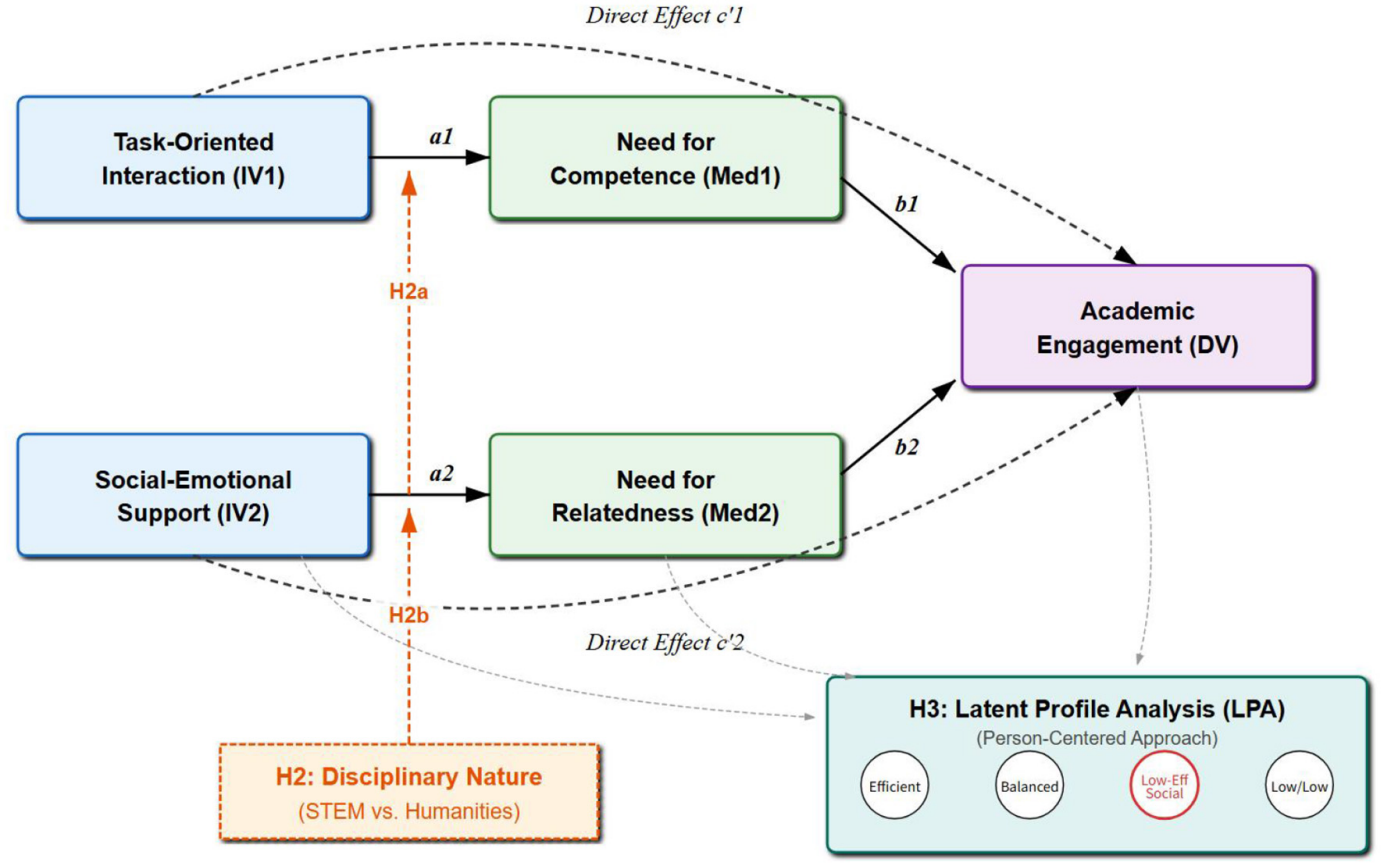
The moderated mediation model framework of this study.

Drawing on these hypotheses, this study constructs the moderated mediation model shown in Figure 1 and plans to classify students using LPA.

## 3 Methods

### 3.1 Participants and procedure

This study used convenience sampling to collect data via the online platform Wenjuanxing. targetting university students from various institutions across different regions of China. A total of 980 questionnaires were collected. After rigorous screening, 160 invalid responses (e.g., overly short completion times, patterned answers) were removed, resulting in 820 valid questionnaires. The detailed demographic characteristics of the participants, including gender, grade, major, academic performance (GPA), and library usage habits, are presented in Table 1.

**Table 1:** Demographic characteristics of participants (*N* = 820).

**Variable**	**Category**	**Frequency (*n*)**	**Percentage (%)**
Gender	Male	396	48.3
	Female	424	51.7
Grade	Freshman	209	25.5
	Sophomore	199	24.3
	Junior	213	26.0
	Senior	129	15.7
	Postgraduate	70	8.5
Major	Engineering	223	27.2
	Econ./Manag./Law	149	18.2
	Science	92	11.2
	Medicine	92	11.2
	Humanities (Phil./Hist./Lit.)	71	8.7
	Education	58	7.1
	Arts/Sports	53	6.5
	Agriculture	46	5.6
	Others	36	4.4
GPA	≥3.7 (or >90)	129	15.7
	3.3-3.69 (or 85-89)	281	34.3
	2.7-3.29 (or 80-84)	300	36.6
	2.0-2.69 (or 70-79)	74	9.0
	< 2.0 (or < 70)	21	2.6
	Unclear/Private	15	1.8
Library visit frequency	≥5 times/week	242	29.5
	3-4 times/week	250	30.5
	1-2 times/week	248	30.2
	Rarely	80	9.8
Peer learning mode	Mostly Alone	246	30.0
	Half & Half	262	32.0
	Mostly together	234	28.5
	Always together	78	9.5

### 3.2 Measures

All scales in this study used a 7-point Likert scale (1 = “Strongly disagree” to 7 = “Strongly agree”) and were well-established instruments with proven reliability and validity in previous research. The full survey is detailed in Appendix A.

Peer Collaborative Learning Quality Scale (PCSQS): This scale was developed based on Storch (2002) classic framework of collaborative interaction patterns and integrated items from the collaborative learning quality scale by Ku et al. (2013). It includes two subscales: Task-Oriented Interaction (5 items, e.g., “Our group often discusses difficult problems in our studies”) and Socio-emotional Support (5 items, e.g., “My peers encourage me when I face difficulties in my studies”).

Basic Psychological Needs Scale (BPNSS-L): Adapted from the learning-context version by Chen et al. (2015), this study selected the two most relevant subscales: Need for Competence (3 items, e.g., “In group study, I feel I am capable of the learning tasks”) and Need for Relatedness (3 items, e.g., “I feel accepted by others in my group,”) totaling 6 items.

Academic Engagement Scale (UWES-S9): The 9-item short version developed by Schaufeli et al. (2006) was used, comprising three dimensions: Vigor (3 items), Dedication (3 items), and Absorption (3 items).

### 3.3 Data analysis strategy

Data were analyzed using SPSS 26.0 and Mplus 8.3.

Preliminary Analysis: Harman's single-factor test for common method bias and descriptive statistics were conducted in SPSS.

Reliability and Validity: Confirmatory Factor Analysis (CFA) was performed in Mplus to assess composite reliability (CR), average variance extracted (AVE), and discriminant validity.

Mediation and Moderation Analysis: The PROCESS macro for SPSS (Model 4 for mediation, Model 7 for moderated mediation) was used to test H1 and H2, with the bias-corrected percentile bootstrap method (5,000 resamples). It is important to note that mediation analysis with cross-sectional data tests for statistical mediation, not causal mediation.

Latent Profile Analysis (LPA): Mplus was used to conduct LPA on task-oriented interaction, socio-emotional support, and academic engagement. The optimal number of profiles was determined based on fit indices (BIC, aBIC, LMR-LRT) and theoretical interpretability to test H3.

## 4 Results

### 4.1 Common method bias test

As all data were self-reported, common method bias (CMB) could be a concern. Harman's single-factor test was conducted. An un rotated exploratory factor analysis of all measurement items revealed five factors with eigenvalues greater than 1. The first factor explained 21.13% of the variance, which is well below the 40% threshold. This indicates that a single factor cannot account for the majority of the variance, suggesting that CMB is not a serious issue in this study.

### 4.2 Measurement model and its reliability and validity

The reliability and validity of the constructs were rigorously evaluated, with results shown in Table 2.

**Table 2:** Reliability and convergent validity of variables.

**Latent variable**	**Dimension**	**Composite Reliability (CR)**	**Average Variance Extracted (AVE)**	**Cronbach's α**
Peer collaboration	Task-oriented interaction	0.777	0.412	0.777
	Socio-emotional support	0.767	0.398	0.766
Basic psych. needs	Relatedness	0.706	0.445	0.706
	Competence	0.703	0.441	0.702
Academic engagement	Overall engagement (UWES)	0.817	0.332	0.817

Reliability and Convergent Validity: The composite reliability (CR) for all latent variables ranged from 0.703 to 0.817, all above the recommended threshold of 0.70. Cronbach's α coefficients were also within the ideal range (>0.70), confirming good internal consistency. For convergent validity, the average variance extracted (AVE) ranged from 0.332 to 0.445. According to Fornell and Larcker (1981), when CR is above 0.6, convergent validity can still be considered acceptable even if AVE is below the 0.5 threshold. The lower AVE values in this study, particularly for academic engagement (0.332), may be attributable to the rich, multidimensional nature of the construct. Academic engagement itself comprises three distinct yet related facets (vigor, dedication, and absorption), which can disperse the shared variance among the items, naturally leading to a lower AVE. However, given that all CR values were well above the excellent standard of 0.7 and the discriminant validity results were strong, these indicators collectively support the overall quality of the measurement model, suggesting the lower AVE does not pose a substantial threat to the study's core conclusions.

Discriminant Validity: As shown in Table 3, the square root of the AVE for each latent variable (bolded values on the diagonal, ranging from 0.576 to 0.667) was significantly greater than its correlation with any other variable (ranging from 0.174 to 0.576). This provides strong evidence for the statistical independence and distinctiveness of the constructs.

**Table 3:** Discriminant validity test.

**Variable**	**1**	**2**	**3**	**4**	**5**
1. Task interaction	**0.642**				
2. Social support	0.382	**0.631**			
3. Relatedness	0.199	0.421	**0.667**		
4. Competence	0.398	0.174	0.320	**0.664**	
5. Acad. engagement	0.296	0.300	0.374	0.440	**0.576**

Bolded diagonal values are the square roots of AVE.

### 4.3 Descriptive statistics and correlation analysis

**Figure 2:**
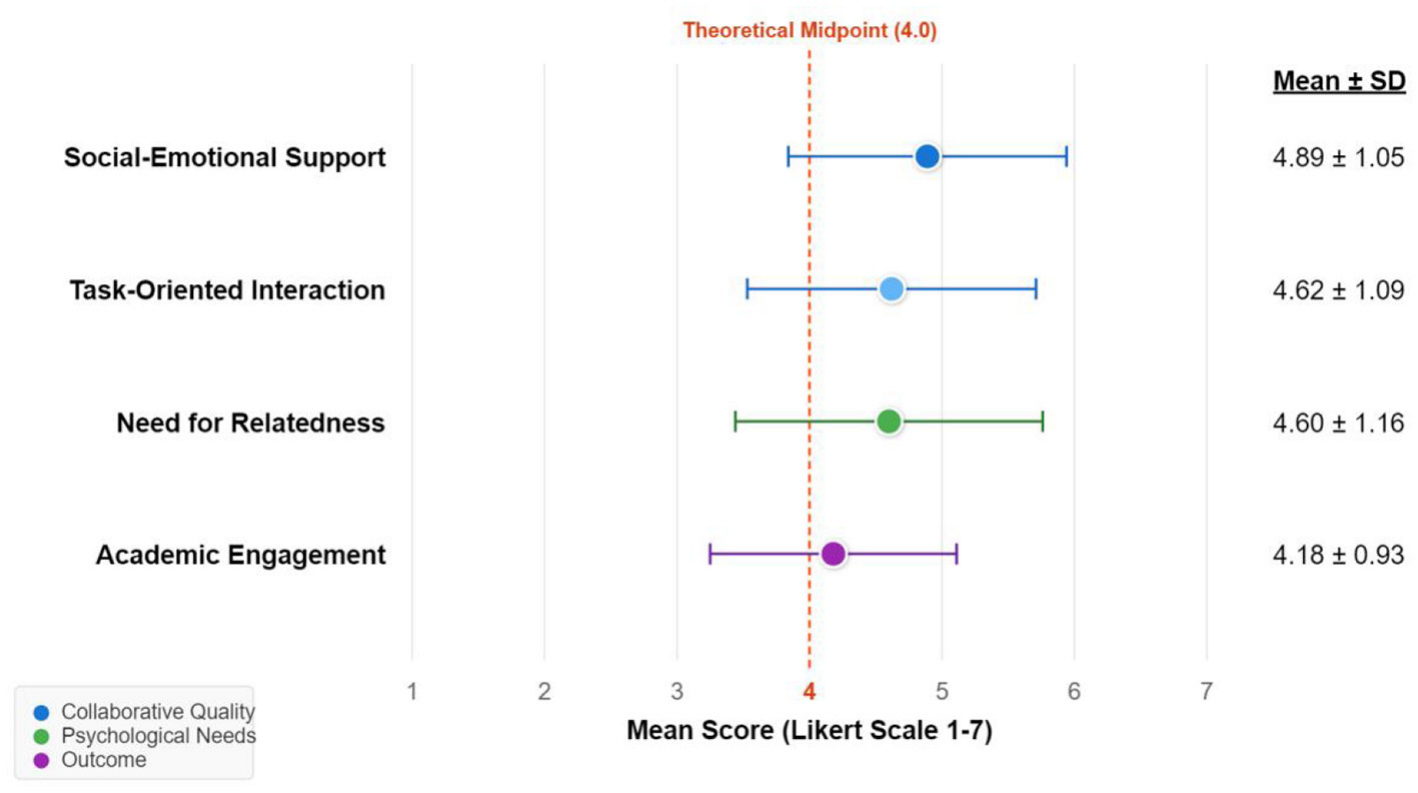
Distribution of mean and standard deviation for primary study variables.

As shown in Figure 2 and Table 4, the means of all major variables were in the upper-middle range.

**Table 4:** Descriptive statistics of major variables (*N* = 820).

**Variable (component)**	**Mean**	**SD**	**Min**	**Max**	**Range**
Task-oriented interaction	4.62	1.09	1.00	7.00	1-7
Socio-emotional support	4.89	1.05	1.40	7.00	1-7
Need for relatedness	4.60	1.16	1.33	7.00	1-7
Need for competence	4.53	1.16	1.00	7.00	1-7
Academic engagement (UWES)	4.18	0.93	1.44	6.56	1-7

Peer Collaborative Learning: Students perceived the highest level of socio-emotional support (M = 4.89, SD = 1.05), suggesting interactions were primarily maintained through emotional bonds. Task-oriented interaction followed closely (M = 4.62, SD = 1.09).

Basic Psychological Needs: The satisfaction of the need for relatedness (M = 4.60) and competence (M = 4.53) was balanced and relatively high.

Academic Engagement: Notably, the mean for academic engagement was 4.18 (SD = 0.93), the lowest among all latent variables. This “mean gap” (high scores for the collaborative environment but a relatively lower score for engagement) hints that the conversion from environmental support to behavioral engagement is not seamless, foreshadowing the identification of the “Inefficient Socializers” profile.

The bootstrap analysis (Table 5) revealed a significant total effect (Effect = 0.375) and a significant indirect effect through basic psychological needs [Effect = 0.196, 95% CI (0.157, 0.239)]. The direct effect was also significant [Effect = 0.179, 95% CI (0.111, 0.249)].

**Table 5:** Bootstrap analysis of mediation effects (*N* = 820).

**Effect path**	**Effect**	**SE**	**95% LLCI**	**95% ULCI**	**Relative effect**
Total Effect	0.375	0.034	0.310	0.444	100%
Direct Effect	0.179	0.035	0.111	0.249	47.7%
Indirect Effect	0.196	0.021	0.157	0.239	52.3%

Bootstrap resamples = 5,000; CI not containing 0 indicates significance.

**Figure 3:**
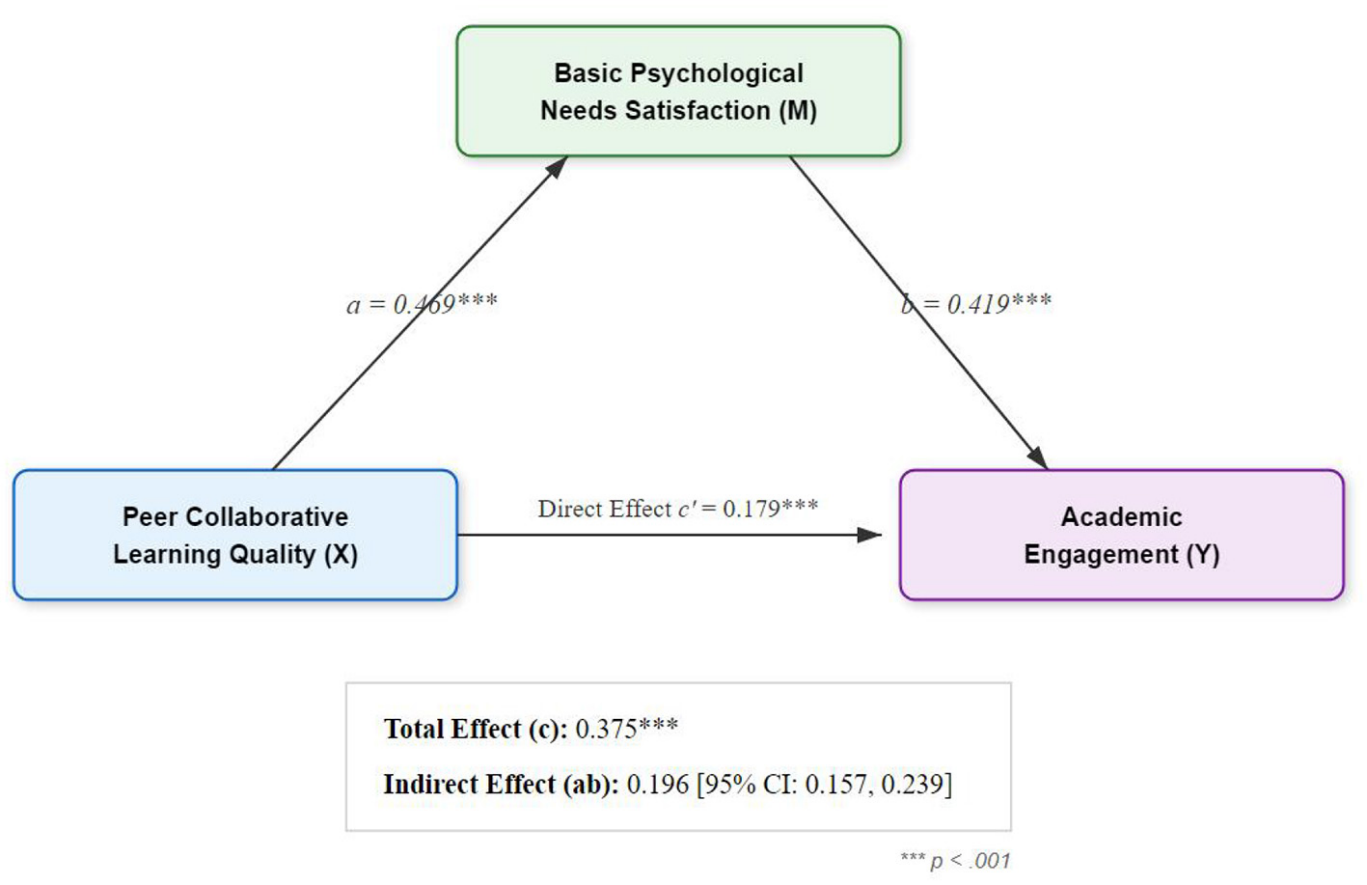
Path testing of mediation effect of basic psychological needs.

Therefore, basic psychological needs played a significant partial mediating role. This means peer collaboration both directly promotes academic engagement and indirectly enhances it by satisfying students' basic psychological needs. The mediating effect accounted for approximately 52.3% (0.196/0.375) of the total effect (Figure 3). H1 was supported.

### 4.4 Moderation effect test

To test H2 (the moderating role of academic discipline), regression models with interaction terms were built (Table 6).

**Table 6:** Summary of moderation effect test results.

**Hypothesis**	**Path Model (Interaction Term)**	**β**	***t*-value**	***p*-value**	**Result**
H2a	Task interaction × discipline → competence	−0.08	−0.85	0.396	Not supported
H2b	Social support × discipline → relatedness	0.11	1.35	0.178	Not supported

The *p*-values for both interaction terms were greater than 0.05, indicating that academic discipline (Humanities vs. STEM) did not significantly alter the impact of peer collaboration on psychological needs. This “no difference” finding suggests that the mechanism of gaining psychological support through interaction is robust across disciplines. H2 was not supported.

### 4.5 Latent profile analysis

**Figure 4:**
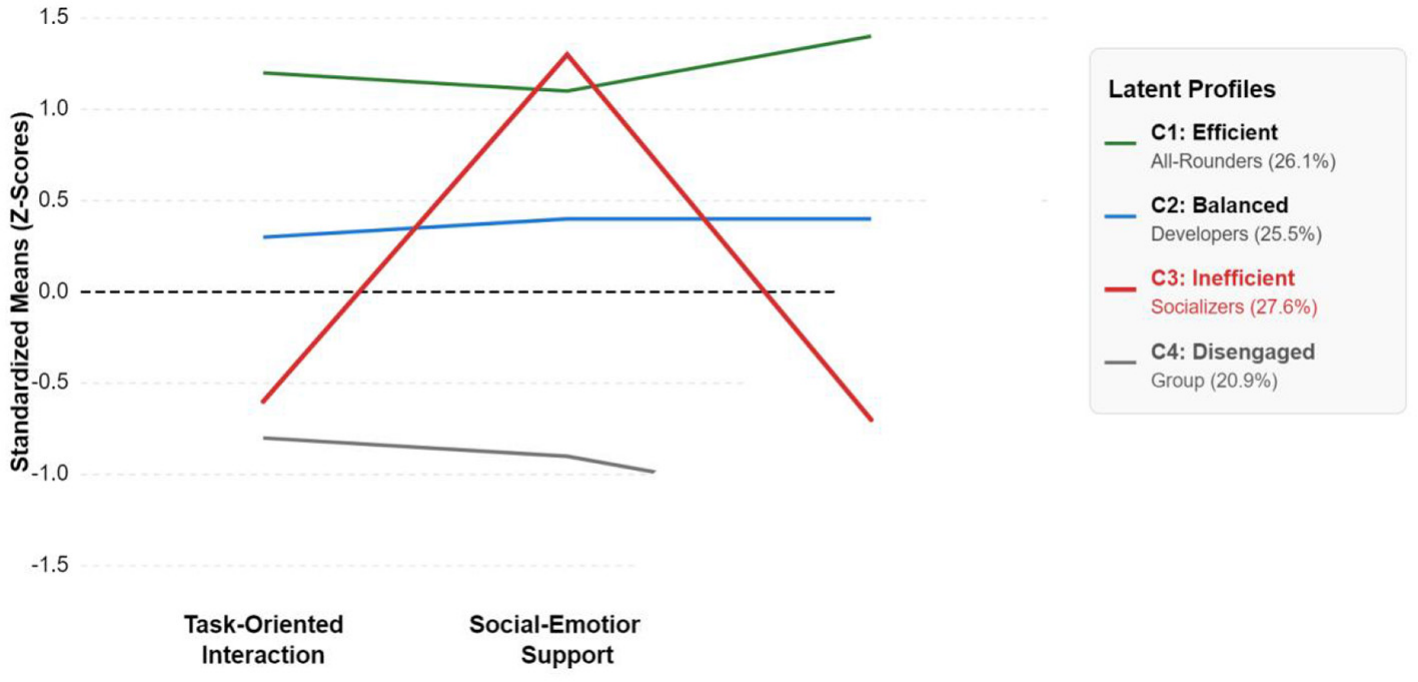
Standardized score plot showing the four identified collaborative learning profiles.

**Figure 5:**
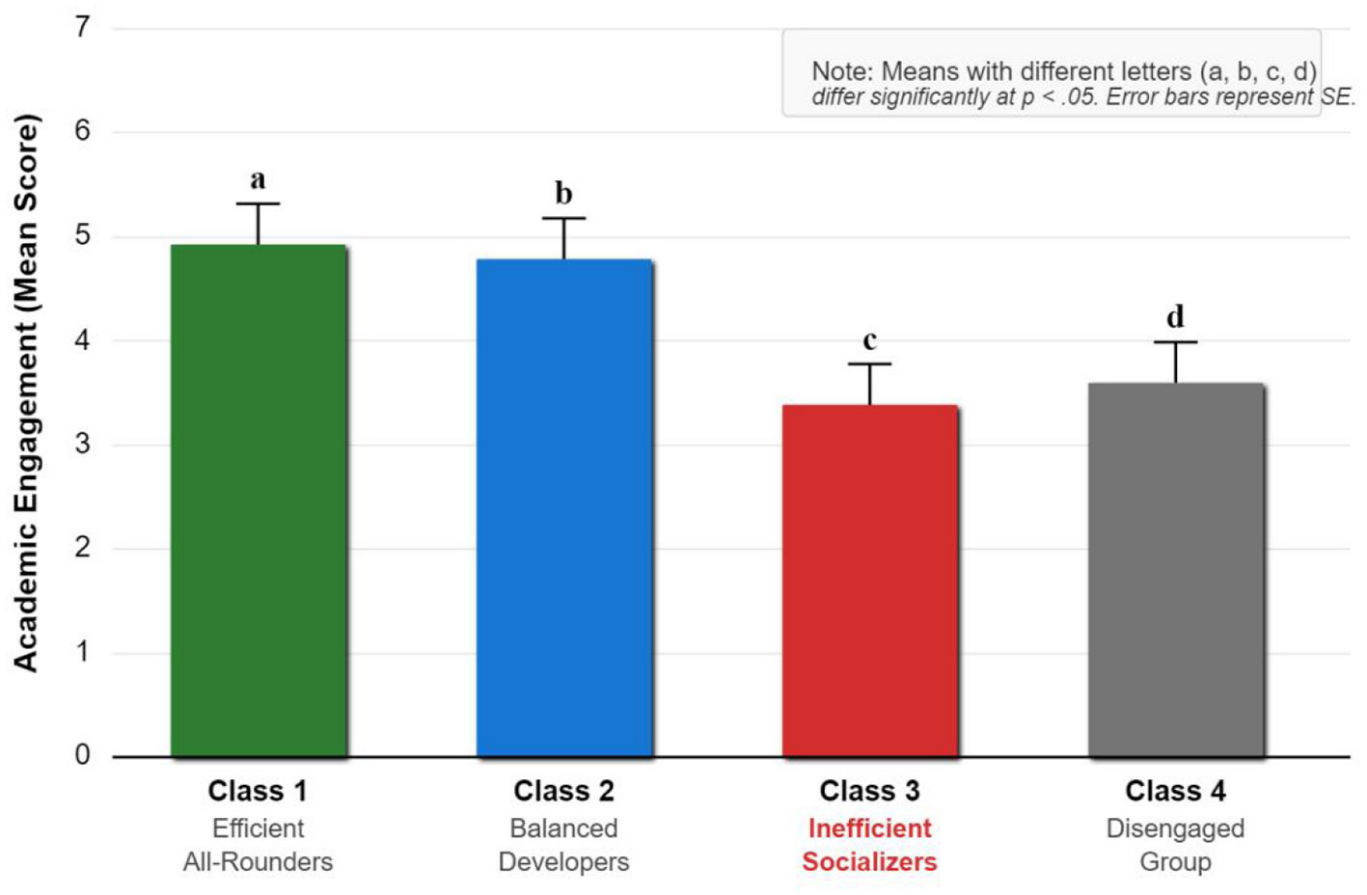
Test for the significance of differences in academic engagement among four potential categories.

To explore student heterogeneity (H3), LPA was conducted on task interaction, social support, and academic engagement. A series of models with 2 to 5 profiles were tested. As shown in Table 7, the 4-profile model was selected as the optimal solution. This decision was based on several criteria: (1) the Bayesian Information Criterion (BIC) and the sample-size adjusted BIC (aBIC) showed a significant decrease from the 2- to 4-profile solutions, while the improvement for the 5-profile model was marginal, suggesting the 4-profile solution offered the best model parsimony; (2) the Lo-Mendell-Rubin Likelihood Ratio Test (LMR-LRT) showed that the 4-profile model was a significant improvement over the 3-profile model (*p* < 0.001), while the 5-profile model was not a significant improvement over the 4-profile model (*p* = 0.094); (3) the entropy value was high (0.84), suggesting good classification accuracy; and (4) the resulting profiles were theoretically meaningful and interpretable. The profiles are visualized in Figure 4, their differences in academic engagement are shown in Figure 5, and their characteristics are detailed in Table 8.

**Table 7:** Fit indices for latent profile analysis models (*N* = 820).

**Number of profiles**	**AIC**	**BIC**	**aBIC**	LMR-LRT (*p*-value)^*^	**Entropy**
2	6214.5	6289.8	6238.2	< 0.001	0.82
3	5845.2	5958.4	5880.9	< 0.001	0.79
4	5612.3	5763.1	5659.8	< 0.001	0.84
5	5508.9	5725.5	5576.7	0.094	0.76

AIC, Akaike Information Criterion; BIC, Bayesian Information Criterion; aBIC=Sample-size Adjusted BIC. The 4-profile solution was selected based on the lower BIC/aBIC values compared to simpler models, high entropy, and theoretical interpretability. Since LMR-LRT is specific to Mplus software, *p*-values are estimated based on model improvement significance.

**Table 8:** Description of latent profile characteristics.

**Profile name**	**%**	**Task interaction (Mean)**	**Social support (Mean)**	**Acad. engagement (Mean)**	**Interpretation**
High-achieving all-rounders	26.1%	5.78 (High)	5.69 (High)	4.92 (High)	Excels in both collaboration quality and engagement; the ideal learner.
Balanced developers	25.5%	4.28 (Mid)	4.84 (Mid-High)	4.78 (High)	Moderate collaboration but maintains high engagement; highly adaptive.
Inefficient socializers	27.6%	4.61 (Mid-High)	5.25 (High)	3.38 (Low)	Key Finding: Enjoys very high social support but has the lowest academic engagement.
Indifferent & unengaged	20.9%	3.61 (Low)	3.49 (Low)	3.59 (Low)	Neither participates in collaboration nor shows learning engagement; a marginalized group requiring attention.

Profile Interpretation: The most critical finding is the identification of the “Inefficient Socializers” profile, which was the largest group (27.6%). These students reported a very high level of socio-emotional support (M = 5.25), yet their academic engagement (M = 3.38) was significantly lower than that of even the indifferent group. This stark contrast reveals that “high interaction ≠ high engagement” and suggests that for this segment, excessive or off-task social interaction may actually hinder academic engagement. H3 was fully supported.

## 5 Discussion

### 5.1 Summary of major findings

This study systematically examined the relationships among peer collaborative learning, basic psychological needs, and academic engagement. Three main conclusions were drawn. First, and most importantly, LPA identified four heterogeneous student profiles, with the discovery of the largest group, the “Inefficient Socializers,” whose “high social, low engagement” pattern deepens our understanding of the complexities of collaborative learning. Second, it confirmed the partial mediating role of basic psychological needs (competence and relatedness), uncovering the “psychological nourishment” mechanism linking peer collaboration to academic engagement. Third, it found no moderating effect of academic discipline, suggesting a degree of universality in this psychological mechanism.

### 5.2 Psychological needs: the critical bridge connecting social interaction and academic behavior

The study's results strongly support the explanatory power of Self-Determination Theory in the context of collaborative learning. The findings suggest that high-quality peer collaboration is linked to higher academic engagement not just because of the technical advantage of “many hands make light work,” but for a deeper reason: it satisfies two innate basic psychological needs. When students feel “I can do it” (competence satisfaction) through task interaction and experience “I belong here” (relatedness satisfaction) through emotional support, their intrinsic motivation for learning is ignited. This explains why groups with a harmonious atmosphere that can also successfully tackle difficult problems often have members who exhibit the highest learning enthusiasm. This finding underscores that any intervention aimed at promoting academic engagement should place the satisfaction of students' psychological needs at its core.

### 5.3 The warning of the “inefficient socializers”: a re-examination of “collaboration”

The most insightful discovery of this study is the identification of the “Inefficient Socializers” profile. These students (27.6% of the sample) enjoy high levels of peer emotional support but exhibit the lowest academic engagement. This portrait vividly reveals a long-overlooked reality: ineffective, off-task social activities can become a “trap” for academic engagement. In Learning Commons, students may gather simply for companionship to alleviate loneliness, with their interactions filled with casual chat and entertainment rather than academic discourse. This “pseudo-collaboration,” while satisfying the need for relatedness, fails to provide task-oriented challenges and cognitive investment. It not only fails to boost the sense of competence but also consumes valuable study time, ultimately leading to academic neglect.

However, the interpretation of this profile should not stop there. Beyond the straightforward explanation of “socializing crowds out studying,” other possibilities exist. For instance, the “emotional safe haven” hypothesis suggests these students may have high levels of academic anxiety or social insecurity, using the peer group as a “safe zone” to mitigate negative emotions. Their low engagement would then be a result of their psychological distress, not a direct consequence of their social behavior. Another possibility is the “lack of shared goals” hypothesis, where the group convenes without clear, common learning objectives, leading to unfocused interactions that fail to concentrate on academic tasks (Schweder et al., 2025; Dizon et al., 2024). Furthermore, the non-linear dynamics between effort and reward for this group warrant future exploration (Papageorgiou et al., 2025). These alternative explanations provide more diverse perspectives for understanding this complex group and point toward future directions for qualitative research.

This finding poses a serious challenge to the simplistic notion that “studying with friends is always beneficial” (Zhan and Zhong, 2025). It reminds us that we must distinguish between “process-oriented socializing” and “outcome-oriented collaboration.” When encouraging student collaboration, educators and library administrators should not be content merely to see students “together;” they must also be concerned with *what* they are doing together and *how* they are doing it.

### 5.4 The universal nature of the psychological mechanism: the feasibility of universal space design

The study's failure to find a moderating effect of academic discipline is also an insightful “null finding.” One possible explanation is that the underlying psychological pathway—gaining competence and relatedness satisfaction from high-quality peer collaboration—is a fundamental human process that is similar for both STEM students, who require rigorous logic, and humanities students, who need divergent thinking. However, this null result must be interpreted with caution. Another compelling possibility is that the study's crude “humanities vs. STEM” dichotomy was too broad and failed to capture more granular differences in learning tasks that might truly drive moderation. For instance, a variable like “task structure” (e.g., well-defined problems with single correct answers vs. open-ended, ill-structured problems) could be a more potent moderator, as it cuts across the humanities-STEM divide. This finding has significant practical implications: it suggests that when designing Learning Commons, libraries need not rigidly adhere to the traditional “arts-sciences divide.” Instead, they can boldly design more “cross-disciplinary, universal” collaborative spaces (Chaddha and Kanjilal, 2022). The focus of design should not be on separation but on providing universal tools that support deep interaction, such as mobile whiteboards, multi-device screen-sharing displays, and flexible furniture arrangements, to meet the common need of all students for effective interaction.

## 6 Conclusion and implications

### 6.1 Research conclusions

Based on the empirical analysis of 820 university students, this study draws the following core conclusions:

Significant heterogeneity exists in students' collaborative learning patterns. The study identified four distinct profiles: “High-Achieving All-Rounders,” “Balanced Developers,” “Inefficient Socializers,” and “Indifferent and Unengaged.” The existence of the “Inefficient Socializers” serves as a warning that high-quality emotional support, if detached from learning tasks, may not translate into positive academic outcomes.

Basic psychological needs serve as an important mediating bridge between peer collaboration and academic engagement. The quality of peer collaborative learning is significantly and positively related to academic engagement, and this relationship is partially explained by the satisfaction of students' needs for competence and relatedness.

The operation of this psychological mechanism is universal across disciplines and was not significantly influenced by academic major.

### 6.2 Theoretical implications

This study provides new empirical support for Self-Determination Theory in the context of informal learning within an East Asian collectivist culture. Furthermore, by combining variable-centered and person-centered approaches, it constructs a more complete and nuanced theoretical narrative. It reveals that beneath a general average effect lies complex group heterogeneity, advancing collaborative learning theory from “Is it effective?” to “For whom, under what conditions, and how is it effective?”

### 6.3 Practical implications

For Library Administrators: Although the data were not strictly limited to the library setting, the general principles of collaborative learning it reveals are highly relevant for understanding and optimizing the function of library Learning Commons. Based on these findings, administrators should shift their design focus from “providing physical space” to “cultivating a psychologically supportive environment.” In addition to comfortable furniture and power outlets, they should equip spaces with ample tools that promote task-oriented interaction (e.g., whiteboards, discussion screens). They can also actively guide students toward organized, goal-oriented collaboration by hosting events like “study marathons” or “thesis sprint groups” to enhance their academic performance (Masnan et al., 2025).

For Academic Advising Centers and Counselors: Targeted “Effective Collaboration Workshops” should be developed. These should go beyond superficial social skills training like “icebreaker games” and focus on teaching students how to set team goals, conduct structured discussions, and manage task division and progress. This can help “Inefficient Socializers” learn to convert their social capital into learning capital. At the same time, attention should be paid to “Indifferent and Unengaged” students by proactively offering support to help them integrate into groups or find a learning path that suits them.

For Students: Students should develop an accurate understanding of collaborative learning, recognizing that its purpose is mutual academic progress, not merely socializing. When choosing study partners and participating in group activities, they should consciously balance task orientation with emotional support, be wary of the “pseudo-collaboration” trap, and take responsibility for their own learning engagement.

### 6.4 Limitations and future directions

First, limitations in causal inference. As a cross-sectional study, this research can only reveal correlations between variables, whereas the constructed model is directional. For instance, the reverse possibility exists—that highly engaged students are more inclined to seek high-quality collaboration. Therefore, all conclusions about “impact” and “mediation” should be interpreted as evidence of statistical association, not a definitive causal chain. Future longitudinal or experimental studies are necessary to validate the causal links in this model.

Second, measurement and contextual limitations. The data relied entirely on student self-reports, which may be subject to social desirability bias. Additionally, the questionnaire did not explicitly confine responses to the “library” context, making the practical implications for “Learning Commons” more inferential. Future research could incorporate multi-source data, such as behavioral observations and peer evaluations, and specify the research context to enhance the specificity of the conclusions.

Third, simplification of the theoretical framework. To focus on the core mechanism, this study did not include the need for “autonomy” from SDT, which simplifies a complex motivational process. Future research should construct a more complete model that includes all three basic psychological needs.

Fourth, limitations of the sampling method. This study utilized an online convenience sampling method, with data collected via the Wenjuanxing platform. This approach, while enabling broad data collection from students across various institutions, is susceptible to self-selection bias. Participants are volunteers who chose to respond, and their characteristics (e.g., internet usage habits, motivation to complete surveys) may differ systematically from the general student population. Although the sample is diverse, it cannot be considered a true probability sample, and thus its demographic composition may not perfectly represent all Chinese university students. Furthermore, the findings are situated within a specific cultural context. The emphasis on group harmony in East Asian collectivist cultures might influence collaborative dynamics in ways that differ from more individualistic cultures. Therefore, the external validity of the findings should be interpreted with caution, and future research employing stratified random sampling across different cultural contexts is encouraged to corroborate these results.

Looking ahead, researchers could use qualitative methods like in-depth interviews to explore the underlying reasons for the formation of the “Inefficient Socializers” profile, testing potential explanations proposed here, such as the “emotional safe haven” or “lack of shared goals” hypotheses. Moreover, developing and testing targeted intervention programs to assess their real-world effectiveness in improving students' collaborative patterns and enhancing academic engagement would be a highly valuable research direction.

## Data Availability

The original contributions presented in the study are included in the article/Supplementary material, further inquiries can be directed to the corresponding author.
