# Supplementary material for: Peer collaborative learning and academic engagement in university libraries: a moderated mediation model and latent profile analysis
Source: Front Psychol. 2026 Jan 16;16:1759026. doi: 10.3389/fpsyg.2025.1759026 (PMC12855537; doi:10.3389/fpsyg.2025.1759026)
Supplement: Supplementary file 1 [file Data_Sheet_1.ZIP › Appendix A.docx]

**Appendix A**

### **A Questionnaire on Learning Behaviors and Experiences in University Libraries**

**Dear Student,**

We are a research team from Huainan Normal University, and we sincerely invite you to participate in an academic study titled “Learning Behaviors and Experiences in University Libraries.”

**1.Purpose of the Study:** This research aims to scientifically understand how university students’ learning methods in the library, especially behaviors related to studying with peers, affect their psychological experiences and level of engagement during the learning process. The findings will provide a theoretical basis for improving university academic support services and optimizing library space design.

**2.Participation Details:** You will be asked to complete an anonymous online questionnaire. The questionnaire includes some basic demographic information (such as your grade level and major), your learning habits in the library, your interactions with study peers, and some of your personal feelings during study. Completing the entire questionnaire is expected to take approximately 10-15 minutes.

**3.Risks and Discomforts:** This study does not involve any sensitive personal information and contains no questions that are likely to cause psychological discomfort. There are no foreseeable risks associated with participating in this study.

**4.Participation and Withdrawal:** Your participation is completely voluntary. You have the right to suspend or withdraw at any stage of the questionnaire without providing any reason, and your decision will not result in any negative consequences for you.

**5.Anonymity and Confidentiality:** This questionnaire collects data in a completely anonymous manner. We will not collect any personally identifiable information, such as your name, student ID, or contact details. All data will be stored in an encrypted format and used solely for statistical analysis and academic publication in this project. We will strictly adhere to the principles of confidentiality and will never disclose any information that could be used to infer your personal identity.

If you have carefully read and understood all the information above and agree to voluntarily participate in this study, please proceed with the questionnaire. Your continuation will be considered as your consent to participate.

**Thank you again for your trust and valuable time!**

###

### **Part One: Basic Information and Learning Habits**

**1. Your Gender:**
A. Male
B. Female

**2. Your Grade Level:**
A. First-year
B. Second-year
C. Third-year
D. Fourth-year
E. Postgraduate

**3. Your Academic Discipline:**
A. Humanities/Philosophy
B. Economics/Management/Law
C. Science
D. Engineering
E. Agriculture
F. Medicine
G. Education
H. Arts/Physical Education
I. Other

**4. Your approximate Grade Point Average (GPA) from the last academic year:** (If you are a first-year student, you can estimate based on your most recent major exam scores)
A. 3.7 or above (or an average score of 90+)
B. 3.3 - 3.69 (or an average score of 85-89)
C. 2.7 - 3.29 (or an average score of 80-84)
D. 2.0 - 2.69 (or an average score of 70-79)
E. Below 2.0 (or an average score below 70)
F. Prefer not to disclose / Unsure

**5. On average, how often do you study in the physical library per week?**
A. Almost never
B. 1-2 times
C. 3-4 times
D. 5 or more times

**6. When you study in the library, what is your typical situation?**
A. Always study alone
*(If you select this option, the survey will end here. Thank you for your participation!)*
B. Mostly study alone, occasionally with peers
C. About half the time alone, half with peers
D. Mostly study with peers
E. Always study with peers
*(If you select B, C, D, or E, please continue with all the following sections)*

### **Part Two: Peer Collaborative Learning Quality Scale (PCSQS)**

Instructions: The following statements describe specific situations when you study with your peers in the library. Please rate how much each statement applies to you based on your true feelings. (1 = Strongly disagree, 4 = Neutral, 7 = Strongly agree)

| Item | Strongly disagree | 2 | 3 | 4 | 5 | 6 | Strongly agree |
| --- | --- | --- | --- | --- | --- | --- | --- |
| Task-Oriented Interaction |  |  |  |  |  |  |  |
| 1. We discuss difficult points from our courses together. | 1 | 2 | 3 | 4 | 5 | 6 | 7 |
| 2. We share our study notes, materials, or problem-solving strategies. | 1 | 2 | 3 | 4 | 5 | 6 | 7 |
| 3. We ask each other questions to check our understanding of the material. | 1 | 2 | 3 | 4 | 5 | 6 | 7 |
| 4. We plan our learning tasks and progress together. | 1 | 2 | 3 | 4 | 5 | 6 | 7 |
| 5. Discussions with my peers inspire new ways of thinking about the study material. | 1 | 2 | 3 | 4 | 5 | 6 | 7 |
| Socio-emotional Support |  |  |  |  |  |  |  |
| 6. Studying with my peers makes the learning process feel less boring and lonely. | 1 | 2 | 3 | 4 | 5 | 6 | 7 |
| 7. When I encounter difficulties or feel frustrated with my studies, my peers give me encouragement and support. | 1 | 2 | 3 | 4 | 5 | 6 | 7 |
| 8. We create a mutually encouraging and positive learning atmosphere. | 1 | 2 | 3 | 4 | 5 | 6 | 7 |
| 9. We supervise each other, reminding one another not to get distracted for long periods (e.g., by using our phones). | 1 | 2 | 3 | 4 | 5 | 6 | 7 |
| 10. I really enjoy the time I spend studying with my current peers. | 1 | 2 | 3 | 4 | 5 | 6 | 7 |

### **Part Three: Learning Experience and Feelings Scale**

#### **A. Academic Engagement Scale (UWES-S9)**

**Instructions:** The following are statements about your feelings while studying. Based on your recent study experiences, please choose the option that best describes your situation. (**1 = Never, 4 = Sometimes, 7 = Always**)

| Item | Never | 2 | 3 | 4 | 5 | 6 | Always |
| --- | --- | --- | --- | --- | --- | --- | --- |
| 1. When I’m studying, I feel bursting with energy. (Vigor) | 1 | 2 | 3 | 4 | 5 | 6 | 7 |
| 2. I am proud of my studies. (Dedication) | 1 | 2 | 3 | 4 | 5 | 6 | 7 |
| 3. Time flies when I’m studying. (Absorption) | 1 | 2 | 3 | 4 | 5 | 6 | 7 |
| 4. In the morning, I feel like going to study. (Vigor) | 1 | 2 | 3 | 4 | 5 | 6 | 7 |
| 5. I am enthusiastic about my studies. (Dedication) | 1 | 2 | 3 | 4 | 5 | 6 | 7 |
| 6. When I am studying intensively, I feel immersed in my work. (Absorption) | 1 | 2 | 3 | 4 | 5 | 6 | 7 |
| 7. I can continue studying for very long periods at a time. (Vigor) | 1 | 2 | 3 | 4 | 5 | 6 | 7 |
| 8. My studies inspire me. (Dedication) | 1 | 2 | 3 | 4 | 5 | 6 | 7 |
| 9. I feel happy when I am studying. (Absorption) | 1 | 2 | 3 | 4 | 5 | 6 | 7 |

#### **B. Basic Psychological Need Satisfaction in Learning Scale (BPNSS-L)**

**Instructions:** Based on your general feelings when studying with peers in the library, please rate how much you agree with each statement. (**1 = Strongly disagree, 4 = Neutral, 7 = Strongly agree**)

| Item | Strongly disagree | 2 | 3 | 4 | 5 | 6 | Strongly agree |
| --- | --- | --- | --- | --- | --- | --- | --- |
| Need for Relatedness |  |  |  |  |  |  |  |
| 1. I feel that I get along well with my study peers. | 1 | 2 | 3 | 4 | 5 | 6 | 7 |
| 2. I care about my peers, and I feel they care about me too. | 1 | 2 | 3 | 4 | 5 | 6 | 7 |
| 3. I feel a sense of belonging when studying with my peers. | 1 | 2 | 3 | 4 | 5 | 6 | 7 |
| Need for Competence |  |  |  |  |  |  |  |
| 4. In the context of studying with my peers, I feel very capable. | 1 | 2 | 3 | 4 | 5 | 6 | 7 |
| 5. Through communication and discussion with my peers, I feel more competent to tackle challenging academic tasks. | 1 | 2 | 3 | 4 | 5 | 6 | 7 |
| 6. I am often able to successfully master the knowledge and skills we study together. | 1 | 2 | 3 | 4 | 5 | 6 | 7 |

**This is the end of the questionnaire.**

We have received your responses. Thank you once again for your valuable time and honest information. Your participation is crucial for the successful completion of this study.

**We wish you academic success and a happy life!**
